# Delivering Therapeutics to the Cochlea: The Importance of the Patient’s Perspective

**DOI:** 10.3389/fncel.2019.00551

**Published:** 2019-12-10

**Authors:** Marie-Josée Duran, Ralph Holme

**Affiliations:** ^1^Fondation Pour l’Audition, Paris, France; ^2^Action on Hearing Loss, London, United Kingdom

**Keywords:** drug delivery, systemic, intratympanic, intracochlear, treatment burden, compliance

## Abstract

Hearing loss represents a major sensory impairment in humans with a strong impact on quality of life. The current standard of care for chronic sensorineural hearing loss is limited to hearing aids and implantable devices like cochlear implants. Treatments for acute hearing loss consist of systemic or intratympanic corticosteroids. Emerging therapies are being developed to prevent hearing loss or to restore it at the cellular level. Many challenges and questions remain as to the delivery of these therapeutics into the inner ear. Scientists, clinicians, and industry stakeholders should always consider the treatment burden from the patient’s perspective when designing new drug delivery approaches. This article highlights key issues to consider.

## Introduction

Worldwide, 466 million individuals suffer from disabling hearing loss and untreated hearing loss is estimated to cost $750bn according to the World Health Organization (WHO) making it a major public health issue (World Health Organization [WHO], 2019). Hearing loss can have a devastating impact on quality of life cutting people off from friends and family leading to isolation and loneliness. Hearing loss is also linked to an increased risk of dementia (Livingston et al., 2017). Besides hearing aids and cochlear implants for chronic hearing loss, and corticosteroids for sudden sensorineural hearing loss (Chandrasekhar et al., 2019), no therapies have been approved by regulatory agencies to prevent or treat hearing loss. Such treatments have the potential to address a growing unmet clinical need and transform the lives of millions of people. The most common form of hearing loss is sensorineural hearing loss, which accounts for 90% of all hearing loss (Cruickshanks et al., 2003). Treating this type of hearing loss will require finding safe and effective methods of delivering drug, gene and cell-based therapies to the inner ear - a complex organ, not easily accessible making it a challenge for drug delivery.

### Emerging Therapeutics for Inner Ear Disorders

A recent review of emerging therapies by Schilder et al. (2019) identified 43 biotech and pharmaceutical companies that are, or have been, engaged in developing over 80 different therapies. Over 20 have already reached clinical trials. Most aim to prevent hearing loss caused by exposure to loud noise, medications that are toxic to the ear, such as aminoglycoside antibiotics or cisplatin, or to treat sudden sensorineural hearing loss. A smaller number aim to restore hearing by triggering the regeneration of damaged cell types or correcting genetic causes of hearing loss by gene therapy. Cell therapies to replace damaged inner ear cells are also under development.

The success of these approaches will require the therapeutic to reach its site of action at the right dose, over the right time course and without causing unwanted side effects. Research into developing techniques to efficiently deliver therapeutics into the inner ear is therefore timely and of the upmost importance. It is also vital to consider what patients want and need from new treatments.

### Therapeutic Delivery: Considerations From the Patient’s Perspective

#### Systemic

The most desirable treatment from the patient’s perspective would be a drug taken orally with no adverse side effects. This will be particularly important for prophylactic treatments targeting age-related hearing loss that may require frequent dosing or for otoprotective drugs designed to be administered before exposure to noise. An oral tablet that can be self-administered would have minimal impact on everyday life and would be the ideal solution for prevention of noise-induced hearing loss amongst military personnel.

Intravenous (IV) delivery of prophylactic treatments may be acceptable to patients if the frequency of dosing is low, for treating acute or sudden onset hearing loss where a patient may be actively seeking medical help, or is already under medical care if being treated with IV aminoglycosides or cisplatin. Regular dosing might require time off work and disruption to everyday life making such treatments less acceptable to patients.

Although systemic delivery of therapeutics to the cochlea is most desirable it may not always be possible. Challenges can include unwanted side effects and, depending on the drug’s chemical properties and pharmacokinetics, insufficient amounts crossing the blood-labyrinth barrier and reaching the cochlea (Salt and Plontke, 2018).

#### Intratympanic

Injection of a therapeutic through the tympanic membrane may overcome some of the challenges faced with systemic delivery of inner ear therapeutics. It may reduce the risk of unwanted side effects and increase the availability of the therapeutic at the side of action, particularly if used in conjunction with polymers to control drug release over a period of time. Although minimally invasive, patients are unlikely to tolerate repeated administration and may become uncompliant. This is because the procedure can be painful, requires patients to take time from their daily activities to visit a clinic and requires them to avoid water entering the ear while the tympanic membrane heals. This could become burdensome. This route of administration is therefore most suited to the treatment of acute forms of hearing loss or the delivery of therapeutics to trigger regeneration of hearing which may be associated with significant improvement in hearing and infrequent administration. Advances in technologies able to reduce the discomfort and pain associated with intratympanic injection could make this approach more acceptable in the future for frequent administration of therapeutics. One such example uses magnetic forces to drive iron nanoparticles that can be linked to a therapeutic into the middle ear and cochlea (Mittal et al., 2019). It is currently in preclinical development. Another way to decrease repeated drug delivery is by prolonging the time the drug is in contact with the round window through the use of hydrogels able to sustain drug release, reviewed by Patel et al. (2019).

#### Intracochlear

Not all therapeutics injected into the middle ear will reach the cochlea at the required concentration, and for cell and gene therapies direct delivery to cochlea via a cochleostomy maybe the only option. This approach requires surgery under a general anesthesia requiring patients to spend time in hospital and recovering from the surgery. Therefore this approach is unlikely to be acceptable to patients for treatments that need frequent administration or for those that provide only modest improvements in hearing. The approach is also associated with the risk of causing permanent damage to any residual hearing, so is unlikely be acceptable to patients with milder forms of hearing loss. The exception may be people with genetic forms of hearing loss that are predicted to lead to a rapid loss of hearing. Intracochlear administration is therefore most suited to the delivery of “single shot” treatments to restore hearing or correct a genetic fault. Some are considering implantable intracochlear drug delivery devices for regular drug infusions to the inner ear. This alternative could be adopted by patients provided the refill chamber is easily accessible without surgery and the time between refills is acceptable.

Finally, cochlear implants are being designed to deliver drugs to the cochlea. Approaches include coating the electrode in biodegradable eluting polymers or cells to release factors that reduce trauma and promote spiral ganglion survival (Roemer et al., 2016; Scheper et al., 2019). These technologies aim to enhance the benefit gained from cochlear implants, so are likely to be acceptable to patients already undergoing cochlear implant surgery.

## Conclusion

A new generation of therapeutics is emerging. It is vital that the field develops safe and efficient methods to deliver these to the inner ear. Disruptive technologies requiring expertise from different fields (pharmacology, formulation, engineering…) are needed. A collaborative and international effort promoting such multidisciplinary research recently emerged with the launch of the International Society of Inner Ear Therapeutics (Schilder et al., 2018). This will benefit from patients’ input.

Depending on the nature of the treatment and expected outcome, different patient populations are likely to tolerate different levels of risk and invasiveness. It is therefore important to seek the target patient population’s view at the early stages of developing a therapeutic to ensure its planned method of delivery is acceptable.

## Author Contributions

Both authors wrote the manuscript.

## Conflict of Interest

The authors declare that the research was conducted in the absence of any commercial or financial relationships that could be construed as a potential conflict of interest.
